# Detection of Communications Channels in VHF Band for Enhanced Maritime Surveillance

**DOI:** 10.3390/s25237258

**Published:** 2025-11-28

**Authors:** André Lopes, Luís Fernandes, Paulo Chaves

**Affiliations:** INOV-Instituto de Engenharia de Sistemas e Computadores Inovação, 1000-029 Lisbon, Portugal; luis.fernandes@inov.pt (L.F.); paulo.chaves@inov.pt (P.C.)

**Keywords:** software-defined radio, SDR, Very High Frequency, VHF, monitoring, spectral analysis, SVM, KNN, machine learning, FFT

## Abstract

This work aimed to develop and evaluate a real-time communication channel detection system in the Very High Frequency (VHF) band using software-defined radio (SDR). For this purpose, an FFT based spectral analyzer with 32,768 points was designed, capable of converting signals from the time domain to the frequency domain, ensuring efficient characterization of a 3 MHz bandwidth with updates every 0.5 s. Three detection algorithms were developed and compared: Energy Detection (ED) and two Waveform-Based Detection methods supported by machine learning models, SVM and KNN. ED stood out for its low computational requirements, suitable for low-cost systems, but had a limited probability of detection ($P_{d}$) at short distances, with zero detection beyond 500 m. KNN showed superior performance at longer distances, achieving 23% $P_{d}$ at 700 m but insufficient for real-time applications. The SVM model proved to be the most effective, achieving a $P_{d}$ of 80% at 1000 m and maintaining a low false positive rate of around 1%. It is concluded that the SVM model is the most suitable for real-time detection systems in the VHF band, offering a balance between accuracy and usability. The extrapolation of the results demonstrates the system’s potential for coverage greater than 2 km with higher-powered marine radios, around 25 W.

## 1. Introduction

Nowadays, countries are more concerned about the security of their borders, whether they are maritime or terrestrial. Because of this, every year these countries’ budgets have more money allocated to defense, and thereby to the security of their populations. On the European continent, several countries share part of their borders with vast bodies of water, such as the Atlantic Ocean or the Mediterranean Sea, and as a result, in recent years, illegal traffic in counterfeit goods, drugs and even illegal immigrants has increased exponentially, causing great overload and fatigue in the security forces that work there [1,2,3,4].

One of the methods used by traffickers to cross the borders is the use of public Very High Frequency (VHF) radio channels. Smugglers exploit these channels not only for communication but also to coordinate actions between their teams on both sides of a border, for example, at sea and shore. Additionally, they use this low-cost solution to issue real-time instructions to their vessels or vehicles, warning those at sea of the movements of the security forces and border guards, allowing all the merchandise to enter undetected into the countries. Because of this technique, the difficulty of confiscating all the cargo and substances by the maritime authorities has only increased [5].

Another situation that complicates the work of the security forces is that the boats carrying out these tasks are actually commercial vessels, registered and easily identifiable in normal situations, and so would never be identified as a vessel involved in clandestine acts [6]. For all the above reasons, these situations have become increasingly common, and with well-organized operations both on the coast and at sea, illegal trafficking has been able to enter countries more easily, demonstrating how VHF can be widely used for this type of operation, as it offers effective long-range coverage at a very low price.

Communication in the VHF band occurs between 156 and 174 MHz and has more than 80 channels, distributed between simplex and duplex channels divided into ship, shore, and ship/shore (according to ITU-R M.1084-5) [7]. So, a channel assigned to ships is simplex, where it is mostly used for transmissions from ships. A coast channel is also simplex, but it is reserved for onshore transmissions, usually used for things like weather reports or safety information. Then there are ship/shore channels, which allow transmission by both vessels and land, and these channels can be simplex or duplex. Each channel is characterized by a unique number and frequency, which makes the channel identification process relatively simple and easy to identify when activated. The channels to be detected are only those related to ship communications, as this is the most logical approach for detecting suspicious activity. Theses channels have a bandwidth of 25 KHz, but in the middle of these primary channels there are intermediate channels with a bandwidth of 12.5 KHz. If channels with lower bandwidth are transmitting, their presence must be taken into account when determining whether the main channels, the ones with 25 kHz bandwidth, are in use. This is one of the situations that requires careful consideration during the design and fine-tuning of the detection system.

Therefore, this study aims to present a solution that can help detect the smuggling problem explained above by constantly monitoring communication channels in the VHF band. An important aspect to consider is the legal, regulatory, and privacy issues surrounding the continuous monitoring of VHF communication channels. This is because indiscriminate interception of radio communications may be restricted by the ITU and national telecommunications regulations and may constitute a violation of privacy. In order to mitigate potential misuse, the proposed system is designed exclusively to detect whether a ship’s communication channel is active, without listening to or analyzing the content of the transmissions. Thus, the data retained is only used for monitoring or analysis purposes, ensuring compliance with legal restrictions and minimizing privacy risks. So, in order to achieve this goal, it is necessary to have equipment that can analyze the entire spectrum associated with the VHF maritime band for ship communications and ship/coast communications, which would help the authorities to prevent the problem more effectively and with better results. This analysis needs to be in real time and on relatively low-cost hardware [8,9], so it could be implemented on a variety of surveillance boats and speedboats, or checkpoints along the coasts of the countries where this situation is most alarming. To meet these requirements, the proposed system uses software-defined radio (SDR) for flexible, wideband monitoring of the VHF spectrum. Signal processing is handled by single-board computers (SBCs), enabling a compact and low-cost setup for mobile or fixed surveillance [10]. The detection approach combines Energy Detection to identify active signals and waveform-based methods using machine learning models to classify transmission types. This combination improves accuracy while keeping resource use low, supporting real-time maritime communication monitoring.

## 2. Technology Review

Over the years, technological evolution has been remarkably rapid and linear, with communication products capable of transmitting over greater distances, increasingly larger datasets, and all of this more efficiently and quickly. However, this increase is not without a cost, as new protocols need to be developed to enable these transmissions, requiring new frequencies to be allocated. The spectrum being a finite place, with the passing of the years and the technological trend never slowing down, it began to suffer from a lack of available frequencies that could be designated for future protocols. As a result, a new area of study emerged, where the main objective was to identify holes, or free spaces in the frequency spectrum, and dynamically and autonomously allocate new transmissions, or, if occupied, find new free slots for them depending on their protocol. This area was designated cognitive radio [11]. Therefore, cognitive radio englobes different sectors of the communications field, from spectrum sensing to spectrum management and even spectrum sharing. To analyze the spectrum in the VHF band and detect active communications, it will be interesting to explore the spectrum sensing sector in greater depth and better understand what types of algorithms and products have been developed over the years, as well as the results that have been achieved. Two of the methods that are most prominent in various articles are the Energy Detection method and the Waveform Detection method, one for its efficiency and simplicity and the other for its intelligence and accuracy, respectively.

### 2.1. Spectrum Sensing

Spectrum sensing is essential for the real-time detection of active maritime communication channels, as it is this method that enables continuous analysis of the radio frequency spectrum [12]. This form of analysis provides insights into the composition of the background noise, the type and characteristics of the signals present, such as their modulations and frequencies, the allocation of these signals in the spectrum, and the free frequencies that can be used for new channels, among other aspects. So, the goal and the methods used highly depend on the application since what may be an excellent result in one context may not be in another. A good example of this is in cognitive radio systems for mobile networks, where the main objective is simply to quickly detect a transmission existing in a given period, while in a military monitoring system, detection involves not only the existence of a transmission but also the type of modulation that this communication employs, as well as the protocol being used since the advantages inherent in discovering this information are far greater than just an elementary detection [13].

There are three types of methods for detecting and analyzing communications: blind, semi-blind, and non-blind. All three methods ensure spectrum monitoring and management, with the key difference between them being prior knowledge of the characteristics of the signal or spectrum to be observed or detected. The blind method, such as the Energy Detection algorithm, does not require any prior knowledge about the signal to be detected. It is based only on measuring the power of the received signal and comparing it to a defined threshold. Even though it is easy to use and has low computational costs, this method is vulnerable to noise and interference, and does not work well in places with a low signal-to-noise ratio (SNR) [14]. The semi-blind method, exemplified by the Cyclostationary Feature Detection algorithm, uses known statistical properties of modulated signals, such as cyclostationarity. These properties allow the distinction between noise and useful signals, even in lower-SNR conditions. While requiring greater computational complexity, this method offers greater robustness in detection and improved performance compared to the blind method [15]. Finally, the non-blind method, such as Waveform Detection algorithms, assumes detailed knowledge of the signal waveform to be detected, as well as prior knowledge of specific patterns or sequences, as preambles. This method allows for highly reliable and accurate detection and is particularly effective in scenarios where the target signals are well defined. However, its applicability is limited to cases where this information is previously available. In recent years, machine learning and deep learning approaches have also been explored in this context, where models are trained with previously labeled signals to recognize specific waveforms, thus offering a new detection paradigm with great potential, especially in complex and dynamic environments [16].

### 2.2. Energy Detection

The first method explored will be Energy Detection (ED). It is the simplest, fastest, and least computationally complex, which is ideal for systems that do not require high precision and use low-cost hardware [17]. This system is one of the most widely used and referenced in studies for blind methods, as this detection mode does not require prior knowledge of the system, that is, before detection occurs, the application does not need to have any knowledge about the signals, noise, or other parameters relevant to detection [18]. In ED, computing the noise floor value is critical for setting an appropriate decision threshold. The threshold is used to distinguish between the presence or absence of a signal based on the energy measured in a given frequency band. However, its effectiveness depends heavily on an accurate estimate of that noise level, which can be challenging in environments with high variability or interference.

This method is typically used in hybrid systems, where it is not the only detection method, as it has certain limitations for signals with a low signal-to-noise ratio (SNR). In article [19], this method is used simultaneously with the Kurtosis Detection method, where after converting the signals to the frequency domain using a 1024 point Fast Fourier Transform (FFT), the average power of the spectrum as a whole is calculated. With these results obtained, and with a threshold value previously defined based on the base noise value, a decision system is applied, where if the average power value is greater than that threshold value, the signal is considered to be active. The Kurtosis method is responsible for signals that the ED considers to be noisy, or non-signals, and performs its own analysis to validate that there is any type of communication. With both systems, it is possible to manage resources and perform a dynamic analysis of the spectrum and signals, taking advantage of both methods.

The ED method has a very low probability of detection ($P_{d}$) for low SNR values, making it a popular method for demonstrations comparing it with other more effective methods as can be seen in article [20], where various spectral analysis techniques for cognitive radios were explored, and the ED method only achieved a detection probability of around 30% for SNR values of −12 dB. In another study [21], the performance of this method was analyzed, both for its PD and its probability of false alarm ($P_{f}$). $P_{f}$ is a crucial factor in signal detection since a high value of this parameter indicates that the selected algorithm has a high probability of indicating non-existent signals as existing, causing the system to malfunction. This article states that when the $P_{f}$ is higher, that is, when the threshold limiting the decision value is lower, the percentage of well-detected PUs is higher, while in cases where the $P_{f}$ is lower, which means that the application is forced to have tighter and more rigorous decision limits, the $P_{d}$ of the primary user (PU) is much lower. One important point to note is that, above a certain threshold, the trend observed up to that point will then begin to reverse.

Lastly, the ED method is especially useful in applications where resources are limited, both in terms of power and money. In article [22], a spectral analysis system was developed using the ED method, where the main goal was to minimize the total implementation cost. Selecting a method such as ED allows these objectives to be reached, as it is a low-complexity algorithm; the choice of both SDR and the computer can be more comprehensive, without requiring high-performance elements, which reduces the cost. In this case, an ADALM Pluto SDR [23] was used, connected to a laptop where the ED algorithm is implemented with great efficiency and over a wide range of frequencies, with good results for the PU detection.

### 2.3. Waveform Detection

The second method to be introduced and analyzed is the Waveform Detection Method, a non-blind method. This method is considered to be more complex and computationally heavier than the previous one, but better results are expected. The two algorithms that will be analyzed with regard to the detection of PU in spectrum sensing, or in the case of this study, the detection of maritime communication channels in the VHF band, are Support Vector Machine (SVM) and K-Nearest Neighbors (KNN) [24]. In order to use both algorithms, it will be necessary to know the waveform of the signal, extracting the associated values, known as features, and then train the models. The values used as characteristics were only the power measurements of the signal waveform. This is because adding phase values as characteristics would increase the processing power required, which would make the system unsuitable for real-time, low-cost operations. However, discarding these values makes the system less reliable and accurate in detecting active communications. Following this, each of them will be presented in detail, along with some of the work that has already been performed and the results that have been achieved [25].

#### 2.3.1. Supported Vector Machine

**Figure 1:**
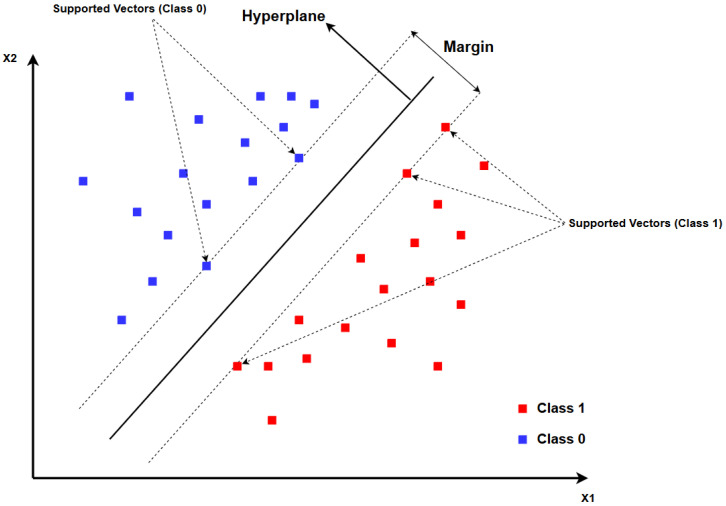
Example of SVM decision model.

The Support Vector Machine, SVM, is one of the algorithms that can be created for the detection of maritime transmissions in the VHF band, using the Waveform-Based Detection method. SVM is a supervised learning method that creates a decision hyperplane to separate data classes in order to maximize the margin between classes as can be seen in Figure 1:

(1) H:w·x+b=0

In the case of two classes, for example, the SVM defines a hyperplane *H*, where w is the vector of weight that determine the hyperplane’s direction, x is the vector of features, and *b* is the bias term, described by Equation (1).

Thus, the *H* hyperplane represents the central line of separation between the two classes. Nevertheless, to ensure that the separation is maximized, the SVM sets two parallel margins to the decisions hyperplane, called $H_{1}$ and $H_{-1}$, with the following equations:

(2) $$\begin{array}{cc} H_{1}\; & :w\cdot x+b=1\\ H_{-1}\; & :w\cdot x+b=-1 \end{array}$$

Theses hyperplanes correspond to the borders of each class’s margins, and the data points closest to the hyperplane, known as support vectors, lie exactly on these margin lines, that is, at $H_{1}$ for one class and at $H_{-1}$ for the other. The distance between the hyperplanes $H_{1}$ and $H_{-1}$ indicates the margin’s width, which is equal to $\frac{2}{∥w∥}$.

In an attempt to maximize the interval between classes, SVM tries to minimize ∥w∥, thereby guaranteeing that all the training points are on the correct side of the hyperplane. This requires that each point $x_{i}$ must satisfy the condition $y_{i}\left(w\cdot x_{i}+b\right)\geq 1$, which places every point outside of the margin zone.

So, the SVM optimization problem is

(3) $$\min_{w,b}\frac{1}{2}{∥w∥}^{2}$$

subject to $y_{i}\left(w\cdot x_{i}+b\right)\geq 1$ for all data points *i*.

After solving this optimization problem, the values of w and *b* that defined the ideal decision hyperplane are obtained. Once these parameters are determined, any new point *x* can be evaluated using the decision function:

(4) f(x)=w·x+b

To determine the class label for this new point, a sign function can be applied, where if f(x)>0 the point is assigned to +1, and if the f(x)<0 it is assigned to −1.

Not all the data is linearly separable, and when this happens, it is important for the SVM to find an approach that allows it to effectively separate classes. In these scenarios, the SVM uses kernel functions, which enable the data to be reorganized into a higher-dimensional space where a linear separation can be found. Because of this, choosing a suitable kernel lets the SVM adjust the decision boundary of the hyperplane, making it capable of dealing with non linear data, where some of the best-known functions are polynomial, RBF, and sigmoid. So with the introduction of kernel functions, the SVM is able to solve more complex problems, selecting boundaries that maximize the margin between classes, even in situations where a linear separation is not possible in its original space.

Several articles and studies have already explored signal/PU detection in spectrum sensing methodology for cognitive radio and real-time monitoring. In articles [26,27], the use of models with SVM shows that performance and detection probability are much higher. For low-SNR conditions [26], the PU and the angle of arrival (AoA) detection was often better in relation to simpler methods such as Energy Detection using multiple antennas. In addition, for a closer analysis of the theoretical use of cognitive radio in detecting holes in the spectrum, or in cases where PUs are not operating on their channels/frequencies, article [27] explores the use of the SVM algorithm. Based on that study, the SVM algorithm performed better than other machine learning algorithms, such as KNN, logistic regression, and decision trees, with real-time PU detection accuracy of around 95%. Another positive aspect of this method is its $P_{d}$ and $P_{f}$ ratio, as it has a high $P_{d}$ and an almost negligible $P_{f}$, with all of this being implemented in real time and with very short decision times.

In a more extensive comparative analysis with other machine learning models, the SVM model once again performed well. In this article [28], three spectrum sensing methods using machine learning algorithms for signal detection were studied, these being Decision Trees, SVM, and KNN. The main objective was to understand the $P_{d}$ and $P_{f}$ levels of each of them, running on a computer and receiving signals through RTL SDR [29]. In each of the algorithms, different types of classifiers were used: five different types were used in SVM and KNN, and three types were explored in Decision Trees. After analyzing the results, it was possible to conclude that SVM and KNN have the best performance with a high probability of detecting the PU and a zero percentage of incorrectly detecting non-existent signals. On the other hand, although the Decision Trees algorithm performed well in detecting PU, as well as the other two algorithms, its $P_{f}$ is higher, which can cause problems for the system.

#### 2.3.2. K-Nearest Neighbors

**Figure 2:**
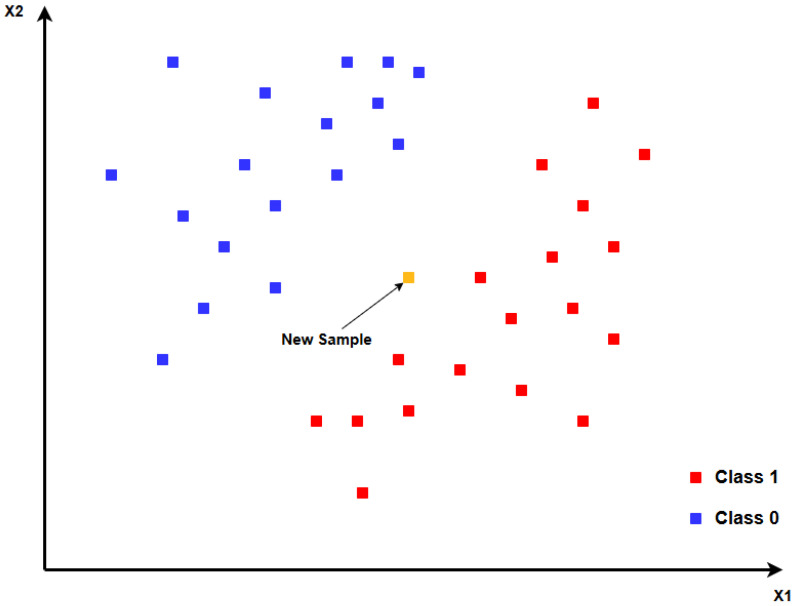
Example of KNN decision model.

The second machine learning algorithm to be analyzed is also supervised learning and is called K-Nearest Neighbors (KNN). This method is one of the most common when using machine learning models, as it is robust and simple to implement. The process of classifying a new sample is based on classes, or sets of its closest neighbors, one example of which is shown in Figure 2.

The task of assigning a class to a new sample invariably begins by calculating the distance between the point to be classified and the training points, which have formerly been labeled. The mathematically expression that lets us find the distance between the two points is presented in Equation (5), referred to as the Euclidean distance, where *x* is the test point, $x_{i}^{\prime }$ are the training points, and *n* is the number of features. A point’s features are measurable attributes of it, which allows us to identify patterns, make previsions and categorize observations:

(5) $$d\left(x,x^{\prime }\right)=\sqrt{\sum_{i=1}^{n}{\left(x_{i}-x_{i}^{\prime }\right)}^{2}}$$

After calculating all the distances between the point subject to classification and the training points, the *K* nearest neighbors are selected, that is, the *K* points in the training dataset that are closest to the sample being analyzed. Once this choice has been made, KNN classifies the point of interest by the predominant class of its neighbors, using the following expression:

(6) $$\hat{y}=arg\max_{c}\sum_{i=1}^{k}\delta \left(y_{i}=c\right)$$

where δ is an indicative function that is 1 if neighbor *i* belongs to class *c*, and 0 otherwise. It is therefore easy to notice that the KNN performance depends on the proper choice of the *K* value since a low *K* can result in the model being susceptible to noise, while a high *K* can hide the differences between classes.

Similar to SVM, there are already several studies on the use of KNN for identifying PU in a certain frequency band, in order to determine whether SU can use that frequency/channel that is temporarily free. KNN can be helpful in these cases, especially in low-SNR situations as described in articles [30,31]. In article [30], the efficiency of this algorithm is analyzed in comparison to more conventional and simplified algorithms such as Energy Detection. KNN is considered quite straightforward and versatile, enabling improved detection capabilities in very low SNR ranges, between −25 and −10 dB. A careful observation of the PU features was made, and when the tests were performed, this model achieved an accuracy of about 94%. In addition, it was possible to verify that this algorithm significantly outperformed the Energy Detection algorithm in low SNR situations, while in higher SNR situations, the performance of both was good. Two aspects that may be considered more worrying, but which do not invalidate the model’s good performance, are the false positives and negatives, which have a performance of 2.5%, and the decision time due to its computational cost, which is considerably slower than ED. In article [31], another crucial factor related to the training and testing of a KNN algorithm was analyzed. This article aimed to understand the best way to optimize the model’s training and testing process, concluding that the best detection probability occurred when 20% of the samples were used for the test set and 80% for the training set. In this parameterization, KNN had an accuracy of 95%.

However, there are other machine learning methods, such as SVM, as described above, and it is important to make a more direct comparison between the performance of both. In this article [32], KNN and SVM were compared for PU detection in the spectrum sensing technique for cognitive radio, where both perform well in detecting signals considered interesting for their applications. One thing that sets them apart, though, is how much computing power each one needs. Since KNN is more mathematically complex, it takes longer to make decisions than SVM, but both are perfect for detecting signals in low SNR environments.

## 3. Spectral Analyzer

The first part of the detection process involves receiving signals in the desired bandwidth and converting all these signals to the frequency domain so that each channel can then be detected. This process corresponds to the creation of a spectral analyzer, which will be implemented using an SDR (BladeRF 2.0 micro XA4 manufactured by Nuand LLC, San Diego, CA, USA) [33], and GNU Radio signal processing modules (GNU Radio Project, USA) [34].

**Figure 3:**
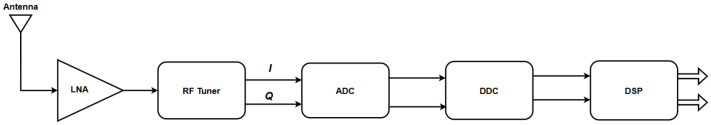
Example of the block diagram for SDR BladeRF 2.0 micro XA4.

The use of SDR is fundamental and essential to achieving the desired objectives, as it allows for the simultaneous analysis of a high range of frequencies, which would not be possible with analog receivers. This is due to the structure of the SDR, as shown in Figure 3, where most of the processing is performed digitally, allowing great flexibility both in the operating range and in the necessary filtering levels and desired amplification. The Low Noise Amplifier (LNA), the RF Tuner and the Analog-to-Digital Converter (ADC) together form the radio frequency front end responsible for selecting, amplifying and converting the incoming RF signal into the digital domain, while the Digital Down Converter (DDC) and the Digital Signal Processing (DSP) modules are where all demodulation, filtering, and signal analysis occur. This process ensures a level of efficiency and precision that analog receivers are not capable of.

**Figure 4:**
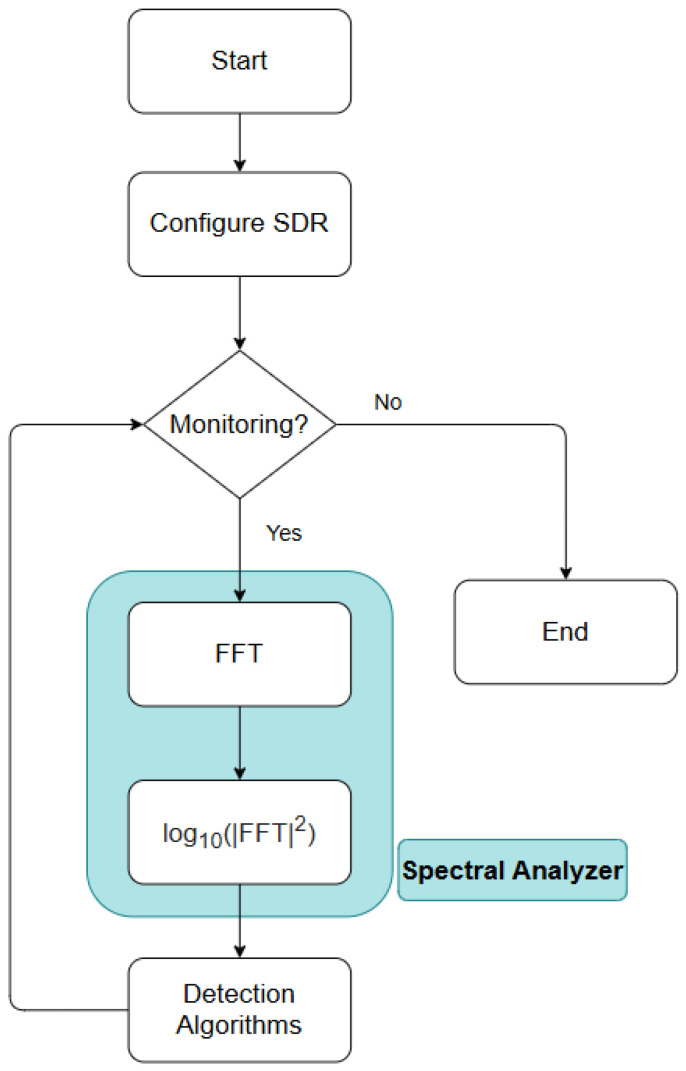
Spectrum analyzer algorithm.

In order to create the spectral analyzer for the constant monitoring of channels in the VHF band, the following algorithm was developed as can be seen in Figure 4. The first step is to ensure the correct configuration of the SDR, using all the parameters essential for detecting these communications. The VHF band is located at the frequencies 156–174 MHz, with the vast majority of ship and ship/coast communications channels being between the frequencies 156 and 158 MHz. It can therefore be concluded that a theoretical bandwidth of 2 MHz is the minimum required to detect all the channels concerned, and that the center frequency should be around 157 MHz, halfway between the upper and lower limits of the band (Equation (7a)). When performing conversions to baseband, the SDR uses a sampling procedure known as IQ Sampling, Quadrature Sampling, or Complex Sampling, which consists of sampling two components: the in-phase component (I), which represents the projection of the signal on the cosine axis (real part), and the quadrature component (Q), which represents the projection of the signal on the sine axis (imaginary part). These two components together represent the signal in complex form, with its representation being I + iQ. Therefore, Complex Sampling can be considered more efficient than real-value sampling since I and Q are acquired simultaneously, which means that together they capture twice as much information. However, Nyquist’s theorem is still satisfied since the two samples per bandwidth are still fulfilled. As a result, theoretically, the minimum required Complex Sampling rate equals the signal bandwidth (Equation (7b)), rather than the higher rate required in the traditional real-valued sampling approach (Equation (7c)).

As a result, the bandwidth and sampling rate chosen were 3 MHz because, due to the SDR internal bandpass filters, a wider bandwidth is required to ensure that there is no filtering in the channels associated with the frequencies at the ends of the band, meaning that the theoretical values calculated above cannot be used. A center frequency of 157.1 MHz was also chosen. The center frequency is slightly offset to avoid a channel at 157 MHz because if it were at that frequency, the DC offsets of the SDR would lead to poorer performance in detecting the channel present at that frequency. Finally, a gain of 20 dB was set for the internal LNA of the SDR in order to have a better signal-to-noise ratio for the entire spectrum under analysis:

(7a) BW=158 MHz−156 MHz=2 MHz

(7b) $$f_{s}=BW\;(\mathrm{IQ}\;\mathrm{Sampling})$$

(7c) $$f_{s}\geq 2\cdot BW\;(\mathrm{Nyquist}\;\mathrm{Sampling})$$

Once the SDR has been properly configured, the monitoring can begin. The transition from time domain signals, which are the type of signals provided by the SDR, to the frequency domain is essential. The main advantage of performing detection in the frequency domain is that when working with multiple frequencies or channels, the efficiency, speed, and ease of detection is much greater because it can isolate and identify each of them, whereas in the time domain, this process is not possible at all. This identification is easily observable thanks to the energy peaks present in the frequencies at which the channels are transmitting, even in cases where the signal strength is weak or low. Another very beneficial aspect of working in this domain is the possibility of observing the base noise value and understanding the noise level that the system is capturing, which then allows for a better understanding of what type of filters and detection techniques will be the best for detecting signals. Baseline noise, also known as white noise, unlike its behavior in the time domain, where it is unstable, in the frequency domain it is uniform and spreads across the entire spectrum that is being analyzed:

(8) $$X\left[k\right]=\sum_{n=0}^{N-1}x\left[n\right]e^{-j\frac{2\pi }{N}kn}$$

To perform this conversion process, it uses one of the best-known techniques, the FFT. The FFT, as represented by Equation (8), will be used to convert the time domain signals collected from the SDR and convert them to the frequency domain. This allows all the advantages listed above to be utilized, creating a much more efficient and simpler detection process. However, the FFT has some configurable parameters, such as the number of desired points. The FFT number of points should have a direct correlation to the resolution level of the spectral analysis, meaning that the more points are available, the more accurate the representation of the spectrum to be analyzed will be. The disadvantage is that more points mean a more mathematically complex system, which invariably leads to a slower system, which can be problematic for a real-time monetization system.

In this case, 32,768 points were chosen on a Raspberry Pi 4B SBC (Raspberry Pi Ltd., Cambridge, UK), a relatively high number, because to cover the 3 MHz bandwidth with multiple channels, fewer points would result in poorly characterized channels, leading to a lower detection rate. While having a higher frequency resolution would improve performance, the bandwidth of the VHF band and of the communication channels is fixed. Therefore, the only viable way to improve resolution was to increase the number of FFT points. However, because GNU Radio (version 3.10.12.0) was used to acquire samples from the SDR, and the FFT is also computed directly on the Raspberry Pi, this necessarily implies a higher processing load. Also, using GNU Radio to both acquire samples from the SDR and compute the FFT directly on the SBC imposes significant processing cost. To compensate for the high number of points, the interval between consecutive FFTs—this is spectrum scans—is reduced, performing spectral analysis every 0.5 s. It is also essential that the SBC maintains a sufficient processing margin to run the detection algorithms for each channel, so further increasing the FFT size or shortening the FFT interval would have a negative impact on the performance of the detection algorithms. Therefore, to ensure that the system was as efficient as possible, several configurations were tested, and the one presented was the one that had the best results in terms of processing speed and obtaining results in real time.

As such, this interval is still more than sufficient for detecting maritime voice transmissions in the VHF band, as voice signals typically last several seconds. The use of a logarithmic scale to represent the signals improves the signal-to-noise ratio analysis and facilitates comparison between different signals, as well as background noise.

## 4. Channel Detection

Next, after the signals have been prepared by the spectral analyzer, channel detection is the following step. Two methods will be analyzed for detection: the Energy Detection Method and the Waveform Detection Method. As mentioned above, only channels related to maritime communications between sea and land will be searched for, analyzed and detected, resulting in a total of 48 channels that need to be evaluated every 0.5 s.

### 4.1. Energy Detection

**Figure 5:**
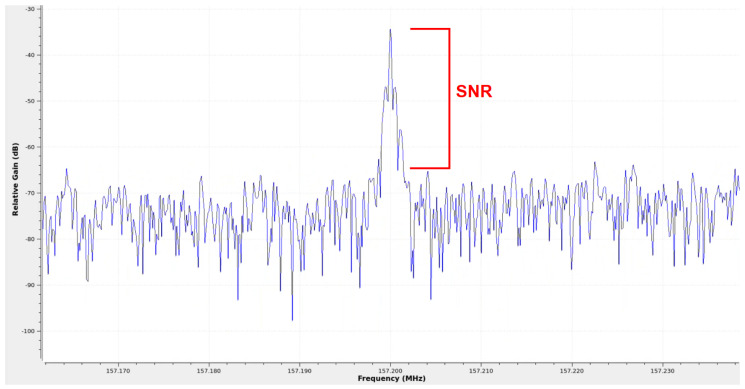
Signal-to-noise ratio (SNR) example.

The first method is the Energy Detection method, which is the simplest to implement and mathematically the least complex. The objective is to calculate the power level of each channel and compare it with a base noise level, and in cases where this occurs, declare the channel as active, as can be seen in Figure 5. In this figure, an active channel at a frequency of 157.20 MHz is shown in the frequency domain after being processed by the spectral analyzer using GNU Radio. Thus, two types of power need to be calculated: noise power and channel power.

The channel power was obtained based on the protocol discussed above, reference [7], where each channel has a bandwidth of 25 KHz. To calculate the power, it is necessary to create a scale factor between the FFT bins and the number of samples produced by the SDR. This factor is called delta, δ. The delta shows how much bandwidth there is between two points of the FFT, which in this module corresponds to 91.55 Hz as shown in Equation (9a). Another important aspect to be calculated is the number of points referring to the bandwidth of each radio communication channel, that is, how each channel of 25 KHz is matched according to the FFT carried out as shown in Equation (9b). In this way, each maritime communication channel in the VHF band will have 273 FFT points characterizing it:

(9a) δ=3 MHz/32768=91.55 Hz

(9b) chBW=25 KHz/δ≈273

The base noise power can be obtained by several methods, ranging from calculating the total average power of the analyzed spectrum to choosing a specific frequency without an assigned channel, creating a channel with the same bandwidth as the communication channels, and then calculating the power value of this fictitious channel. The last method was chosen because it had the fewest disadvantages, since if several channels are active at the same time, the average noise value obtained could be considerably higher, which, for this decision method, would mean that some of the signals with lower power would be discarded due to the lower signal-to-noise ratio. Therefore, the center frequency of the noise channel was set to 155.9 MHz, as it is outside the communication band of maritime VHF channels and is therefore not used for transmission. However, it is still within the RF bandwidth captured by the SDR, and has exactly the same gain, noise figure, and environmental conditions as the communication channels analyzed. This ensures that there is a true measurement of the dominant ambient noise, while ensuring that it cannot be occupied. Something else to consider is that the baseline noise power can vary from case to case. This is because the receiver in the SDR is sensitive to the ambient temperature, and in a warmer environment, the power value will be higher than in a colder environment. In addition, elements such as the LNA, antenna gain, and antenna height can negatively affect this value.

**Figure 6:**
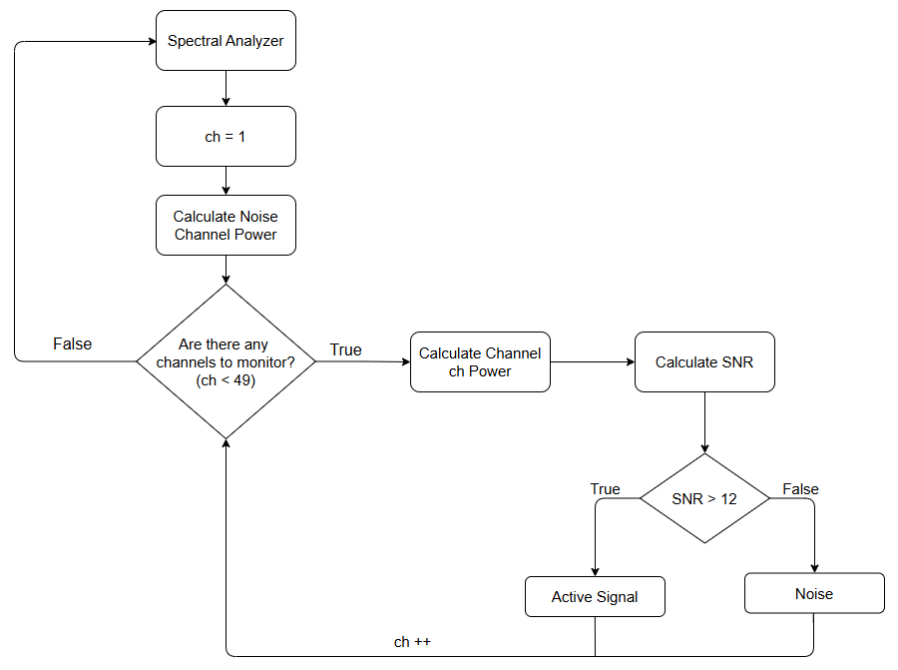
Energy detection algorithm. The notation “ch++” indicates that the channel index is incremented by one.

As can be seen in Figure 6, the first step for each new piece of data provided by the spectral analyzer is to calculate the base noise power. The base noise power is always calculated when a new FFT is generated and used as a reference to calculate the SNR value for each communication channel. The procedure for calculating the noise power is the same as for calculating the channel power.

After calculating the noise power level, it is necessary to define a list with the frequency of each of the 48 channels to be monitored so that the algorithm can loop through it. For each channel, the point corresponding to the channel’s center frequency in the FFT value vector is calculated. Then, each of the frequencies that compose the channel’s bandwidth is added together, and once the sum is obtained, the total power value of the respective channel is obtained. With the total power of the communication channel and the noise, it is possible to find the SNR of the channel, Equation (10), by subtracting the total power value of the channel from the total power value of the noise. If the SNR value is greater than 12 dB, the channel is considered active; otherwise, it is considered inactive or noisy (Equation (11)). The 12 dB threshold is not arbitrary; it comes from the VHF maritime protocol, which specifies a minimum required signal-to-noise and distortion ratio (SINAD) of 12 dB for reliable voice intelligibility on low-power channels [7]. In practical terms, this corresponds to a signal approximately 15.85 times stronger than the noise and defines the minimum SNR at which a channel is considered usable by a VHF receiver. Although a lower limit could, theoretically, be used exclusively for energy-based channel activity detection, the ideal value would depend heavily on receiver sensitivity and environmental noise conditions. Therefore, the minimum intelligibility requirement of the standard, SINAD of 12 dB, was chosen as a reference. This process is repeated for each of the 48 channels:

(10) $$SNR=P_{ch}-P_{noise}$$

(11) $$SNR=\left\{\begin{array}{c} Active:SNR⩾12\\ Noise:SNR<12 \end{array}\right.$$

### 4.2. Waveform Detection

**Figure 7:**
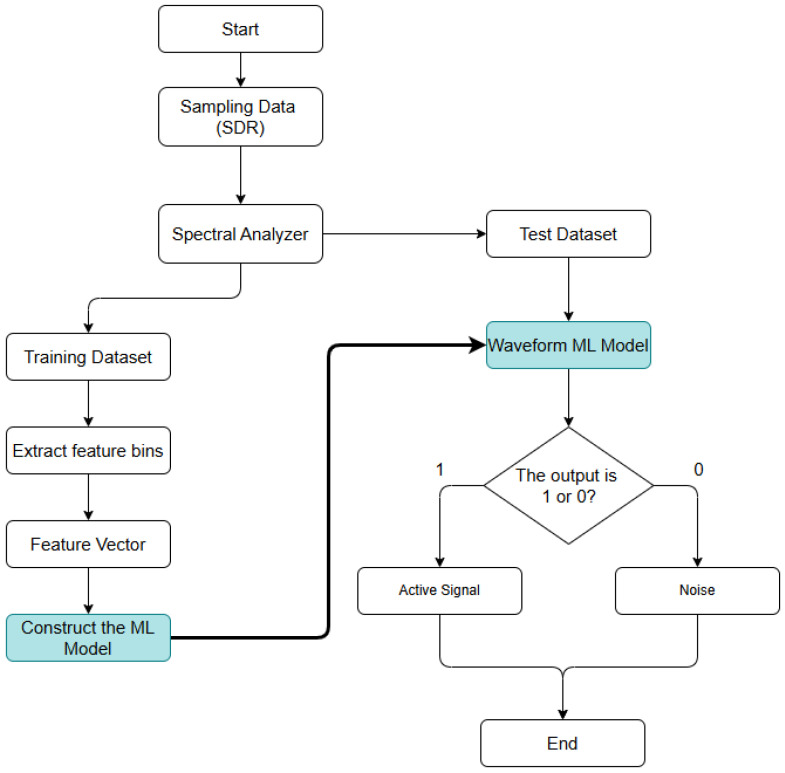
Machine learning models creation algorithm.

The second method consists of using prior knowledge of the signal waveform it is intended to identify, a method known as Waveform-Based Detection. Once the data from the spectral analyzer has been obtained, it is necessary to filter the waveform of an active channel, thus ignoring the rest of the spectrum, creating the conditions for training the models so that they recognize the active channels and make detection more immune to external noise sources. Each communication channel in the VHF band has 273 points associated with it, corresponding to the channel bandwidth, as shown in Equation (9b) above. These points are called features, which are values that describe the behavior of the channel, used by machine learning models to understand the conditions surrounding that channel. With these features, it is possible to develop models, creating feature vectors corresponding to a communication channel as shown in Figure 7. This process relates to model learning, with the second part corresponding to verification, where the datasets for each model are different.

The first part, learning, is when the model is trained based on a set of data previously obtained from the situation to be explored, in this case maritime communication channels in the VHF band. For this reason, data collection was carried out in a large park located next to the river, chosen for its open areas that minimize signal obstruction, while also reflecting realistic conditions for radio propagation. The transmitter used was a 5 W radio. To evaluate the signal-to-noise ratio under varying conditions, three distances were tested: 200, 400, and 500 m. These distances were specifically selected to produce high-, medium-, and low-SNR scenarios. Although VHF radios are normally capable of longer communication distances under ideal conditions, the park environment, low antenna height, and other line-of-sight limitations reduce signal quality, making it significantly worse than what would be achieved with a radio on a ship, where the antenna height would be adequate and the surrounding environment would also be favorable. Nonetheless, these shorter distances are sufficient to generate the range of SNR conditions required for this study. For each of these, the features of the tested communication channel were collected, and feature vectors for the channel with a bandwidth of 25 KHz were created. The data obtained were divided into two datasets: training and testing datasets. The collected data were divided as follows: 90% are for learning and the rest for verification, as it is important to have a large dataset for the models to learn about all the noise situations that may arise and therefore be less vulnerable. To achieve the best performance, two machine learning algorithms were explored: Support Vector Machine (SVM) and K-Nearest Neighbors (KNN). In the learning process for each of the models, various parameters were adjusted to better identify patterns in the data provided.

During the verification process, the testing dataset was used, as can be seen in Figure 7. The objective is to understand how the models will behave with data that they have never encountered before. In this process, a confusion matrix is generated to evaluate the performance of the trained model. This matrix consists of four main values:

- True Positive (TP): Number of cases in which the model correctly predicted the correct class.
- True Negative (TN): Number of cases in which the model correctly predicted another class.
- False Positive (FP): Number of cases in which the model incorrectly predicted the correct class.
- False Negative (FN): Number of cases in which the model incorrectly predicted another class.

These values can be used to calculate different evaluation metrics, such as accuracy, precision, recall, and F1-score. Accuracy (Equation (12a)) calculates the proportion of correct predictions relative to the total number of predictions made. Precision (Equation (12b)) measures the proportion of correct predictions relative to the total number of predictions made by the model. Recall (Equation (12c)) deduces the size of the correct cases identified by the model. The last metric is the F1-score (Equation (12d)), which evaluates the harmonic mean between precision and recall:

(12a) $$\mathrm{Accuracy}=\frac{\mathrm{TP}+\mathrm{TN}}{\mathrm{TP}+\mathrm{TN}+\mathrm{FP}+\mathrm{FN}}$$

(12b) $$\mathrm{Precision}=\frac{\mathrm{TP}}{\mathrm{TP}+\mathrm{FP}}$$

(12c) $$\mathrm{Recall}=\frac{\mathrm{TP}}{\mathrm{TP}+\mathrm{FN}}$$

(12d) $$F1\text{-}\mathrm{Score}=2\times \frac{\mathrm{Precision}\times \mathrm{Recall}}{\mathrm{Precision}+\mathrm{Recall}}$$

**Figure 8:**
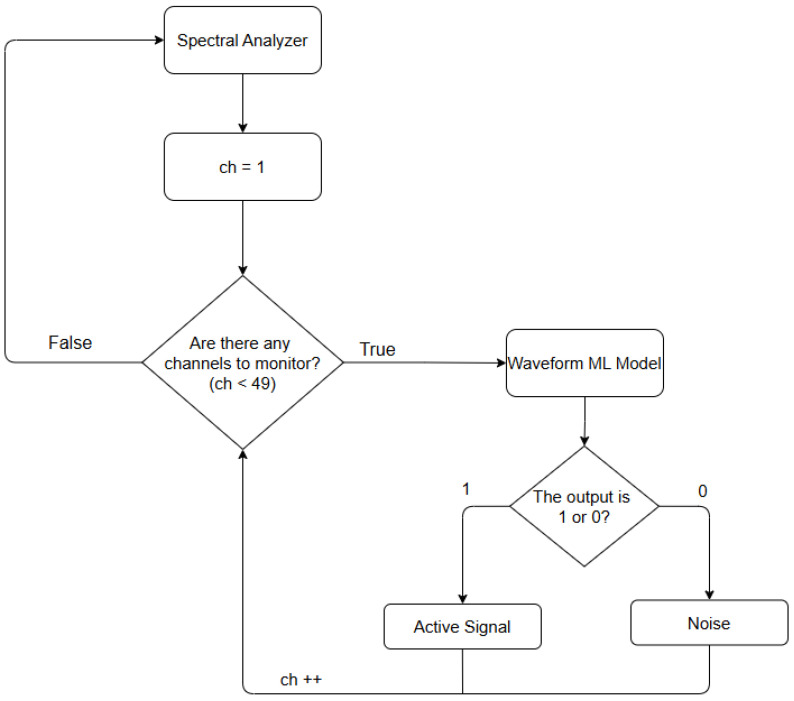
Waveform Detection algorithm. The notation “ch++” indicates that the channel index is incremented by one.

Finally, an algorithm was created to detect active channels in real time using machine learning models as shown in Figure 8. This method collects the characteristics of each channel after the spectral analyzer using an approach very similar to the signal-to-noise ratio model and the learning process, combining the two. These characteristics of each channel are then entered into the models, allowing them to classify these channels as active or inactive. The creation of the two models, KNN and SVM, and the choice of parameters for both will be discussed below.

#### 4.2.1. Supported Vector Machine

**Figure 9:**
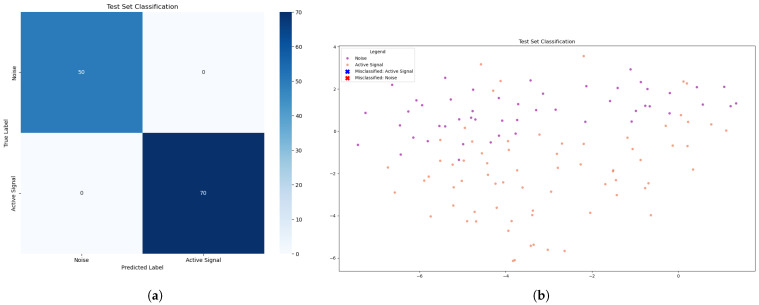
(**a**) Evaluation with the confusion matrix of the best SVM model. (**b**) Visualization of the best SVM model predictions using t-SNE.

In the SVM algorithm, the learning process allowed the generation of a model with 100% detection performance, as can be seen in Table 1. In terms of both accuracy and precision, the model was able to correctly identify the active channel at all times during transmission, without ever producing false positives or negatives. Figure 9a clearly shows the 70 samples when the active channel was active and the 50 moments when it was off. Figure 9b demonstrates this more visually using the t-SNE method.

In order to achieve these results, the model was configured with the following parameters: a second-degree polynomial kernel was used, with gamma defined as scale, coefficient “coef0” equal to 7, and regularization parameter “C” defined as 0.1. The second-degree polynomial kernel allows learning from nonlinear relationships between variables. The gamma parameter, when defined as scale, automatically adjusts based on the variance of the data, affecting the construction of the model. “Coef0” controls the impact of the independent term on the kernel, and because it is a higher value, it increases the complexity of the decision boundary, the hyperplane. Finally, the “C” parameter establishes the balance between maximizing the margin and minimizing the classification error, where a low value, such as 0.1, favors a larger margin, allowing for greater generalization of the model.

#### 4.2.2. K-Nearest Neighbors

**Figure 10:**
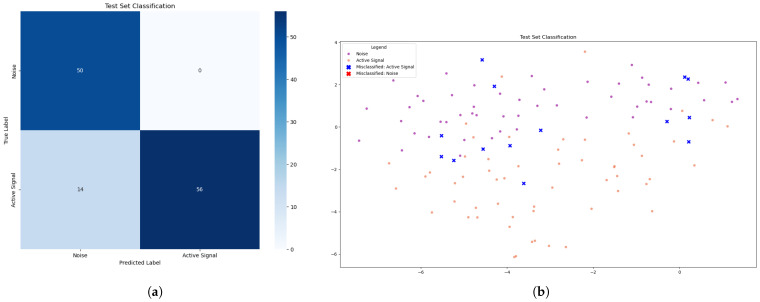
(**a**) Evaluation with the confusion matrix of the best KNN model. (**b**) Visualization of the best KNN model predictions using t-SNE.

In the KNN algorithm, the learning process generated a model where the best possible performance achieved an accuracy of 88%, as well as an active channel detection probability of 80%, with problems also related to false positives as can be seen in Table 2. Figure 10a shows the table with the test results and how the sample was classified according to the model, followed by a visual representation using the t-SNE method; see Figure 10b.

The model developed to obtain these results was configured with 30 neighbors, using distance-based weighting, in which the closest points have greater influence on the classification decision. The use of 30 neighbors allows for a more robust decision, smoothening the impact of characteristics that are less relevant for detecting active channels. Weighting by distance ensures that samples closer to the point to be classified have greater weight in the decision, which contributes to a more sensitive decision boundary to the data distribution, resulting in the model performing better in areas where sample density is higher, such as situations with low SNR.

## 5. Results and Discussion

Following the creation of all algorithms and their models, it is important to conduct a series of tests to verify their accuracy and quality. Therefore, to ensure that the models were well developed and capable of learning from the samples initially collected for this purpose, performance tests were carried out for longer distances, specifically 700, 800, 900, and 1000 m. These tests were performed at the same location where the data for the creation of the models was gathered, due to the conditions of the location. These conditions, an open park environment rather than an open sea environment, have the advantage of providing controlled, repeatable, and easily accessible conditions, a feature deliberately chosen to analyze signals under different signal-to-noise ratios, while not losing the aspect of free propagation. Nevertheless, in an open sea environment, propagation conditions are different, mainly due to atmospheric phenomena such as enhanced signal strength (ESS) and super-refraction effects, which considerably expand the detection range [35]. Despite this, the principles of VHF propagation and the methodology applied here remain relevant and valid for evaluating and modeling the performance of communications detection in this band.

This study is expected to simulate the detection of illegal vessels at multiple distances, providing an understanding of the maximum range for each detection method: Energy Detection, Waveform-Based Detection with SVM, and Waveform-Based Detection with KNN. To ensure variability in the tests, a crucial factor for analyzing results with machine learning models, two different channels, that is, two different frequencies, were always tested at each distance. By testing at multiple distances and on different channels than those for which the models were developed, it is possible to more accurately evaluate the performance and check for overfitting. These analyses are essential to ensure reliable detection of maritime communications in the VHF band, ensuring the reliability and effectiveness of the models and algorithms.

### 5.1. Energy Detection Method

**Figure 11:**
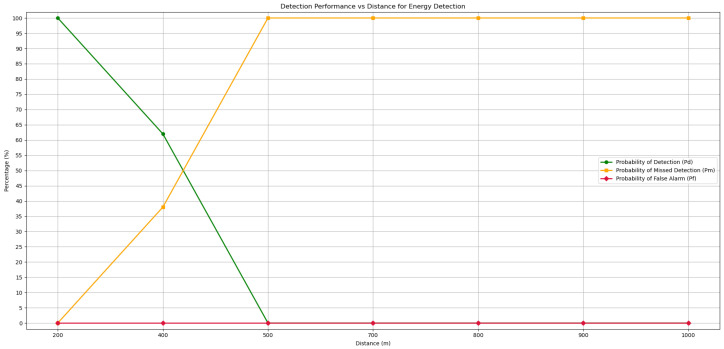
Detection and error probabilities of the Energy Detection algorithm for different test distances.

The first method to be tested was the Energy Detection method. Although this algorithm did not involve any learning system, it was tested at all distances, which are 200, 400, 500, 700, 800, 900, and 1000 m. In all of them, the SNR of the two channels being checked was calculated, and if the value was higher than 12, the channel was considered active as explained in the development of the algorithm. In Figure 11, you can see the performance of the Energy Detection method over these different distances. For each distance, an average of the confusion matrix of the two channels tested was calculated in order to unify the results for each range. This figure shows the $P_{d}$ over the testing distances used in this method, which leads to the conclusion that for relatively short ranges of 200 m, the method performs very well, with a detection probability of 100%. However, when the distance increases slightly, leading to a decrease in the channel’s signal-to-noise ratio, performance begins to drop sharply, with only 62% $P_{d}$ at 400 m and no transmission detected on either channel at 500 m. Performance never improved beyond 500 m.

For detecting false positives, which are $P_{f}$ situations, this method proved to be extremely effective, with a probability of 0% for all distances tested. It means the algorithm showed total accuracy in preventing the absence of transmission from being mistaken for activity on the channel. Such errors usually occur when the SNR is close to the detection threshold, which can lead to false positives. In contrast to the $P_{d}$ probability, the probability of missed detection, $P_{m}$, shows the probability of errors when detecting channel transmissions. Finally, to conclude the results analysis of this detection method, the overall accuracy for a distance of 200 m is 100%, followed by 81% for 400 m, and from there on 50% since communication inactivity moments are always well identified, contrasting with channel activity moments. As such, the Energy Detection method proves useful for detecting communications in the VHF band but only for short distances.

### 5.2. Waveform Detection Method with SVM

**Figure 12:**
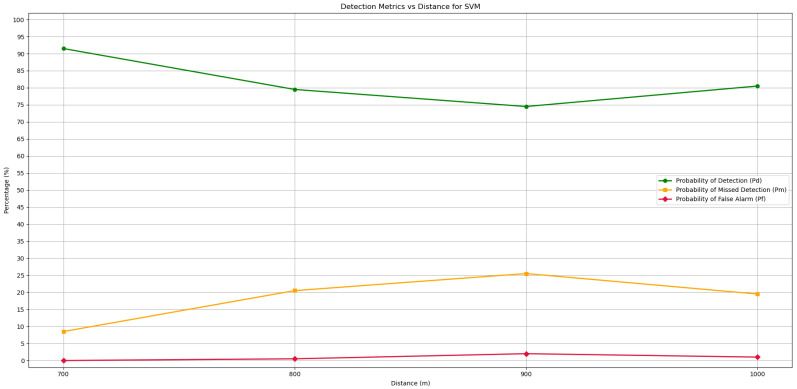
Detection and error probabilities of the Waveform-Based Detection algorithm for the SVM model for different test distances.

The second method tested was the Waveform-Based Detection method with the SVM algorithm. Contrary to the ED method, only the longest distances were used for the performance test, as the shortest distances were used to create the model. The main objective is to understand whether the model created has learning capability, and for this purpose, it was tested up to twice the maximum distance at which the transmission samples were collected, resulting in a maximum tested range of 1000 m. In Figure 12, $P_{d}$, $P_{f}$, and $P_{m}$ are represented for each range, 700, 800, 900, and 1000 m. However, to obtain the results of these parameters, the average performance values of the two channels were calculated for each distance, corresponding to the confusion matrix obtained as was performed in the previous method. The model developed using SVM presents many positive results, with the ability to detect transmissions with an effectiveness of around 80% at 1000 m, considering that under these conditions, the SNR is lower than in the transmissions used for learning at 500 m, showing a clear ability to detect communication channels in the VHF band and for learning. An interesting aspect of the model’s $P_{d}$ is that it can be sensitive to external noise propagation conditions that negatively affect signal quality, as can be seen in the $P_{d}$ at a distance of 900 m, which is lower than that at 1000 m, by around 5%, due to the loss of signal quality received by the SDR. In relation to $P_{f}$, false positives, the SVM model also shows very good results, with probabilities of around 1% for the four distances tested, which means that the user will not receive information about channels that are active when they are not actually active on the system.

Another aspect worth considering is the accuracy of the model at each distance. Accuracy provides a general understanding of the model’s behavior, both in terms of predicting communication detections and distinguishing communication from other noise situations. The performance is quite good for any distance tested, and the predictable behavior of performance loss with increasing distance is not completely noticeable. Despite the shortest distance tested being 700 m, with an accuracy of 95%, the accuracy for 900 m is 86% and for 800 and 1000 m is 90%, which demonstrates a certain stabilization in performance even though the SNR is deteriorating, which is an excellent sign. Therefore, this algorithm proves to be very effective, with excellent results for real-time monitoring situations.

### 5.3. Waveform Detection Method with KNN

**Figure 13:**
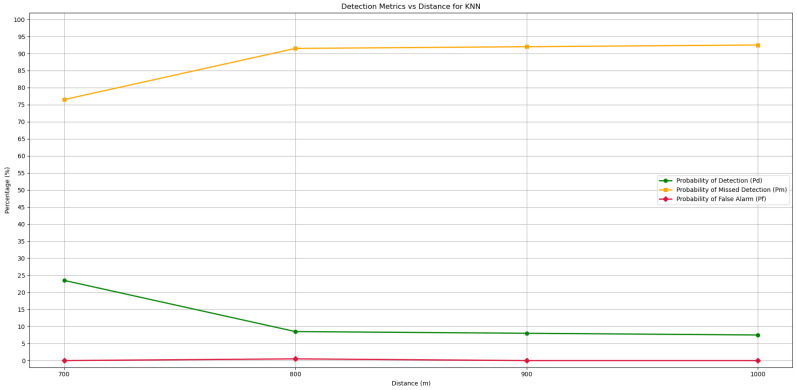
Detection and error probabilities of the Waveform-Based Detection algorithm for the KNN model for different test distances.

The KNN algorithm for the Waveform-Based Detection method was the last algorithm to be tested, and as in the previous machine learning case, it was tested for the same distances. In Figure 13, the four distances tested and the $P_{d}$ for each of them can be seen, as well as the $P_{m}$ and $P_{f}$. Once again, the method of averaging the values of the confusion matrix was used to unify the channels over the same distance. The $P_{d}$ with this type of machine learning model fell short of what was expected, with a very low $P_{d}$ of around 8% for 800, 900, and 1000 m, which means that up to 92% of the transmissions made, $P_{m}$, could not be detected by this model. At a closer distance to the radio transmitter, at 700 m, despite $P_{d}$ being 23%, this value remains very low for a system that needs to detect relatively short voice communications. Regarding $P_{f}$, performance is excellent since the KNN-based model practically never classified moments when the channel was inactive as transmission, regardless of distance.

Overall, the model’s accuracy was found to be relatively low, at around 54% for the longest distances tested and 62% for 700 m. This result is due to the fact that the model performs well only in detecting moments when the channel is inactive, while the detection of active transmissions is not very accurate. Thus, the Waveform-Based Detection algorithm using KNN did not prove to be a suitable solution for the real-time detection of maritime communications at any of the distances studied.

## 6. Conclusions

Given these results, it is possible to draw some interesting conclusions about the three algorithms developed and their performance in a real-time system, as well as about the spectral analyzer created for this purpose. The first objective was to develop a spectral analyzer to convert the signals received from the time domain, as processed by SDR, to the frequency domain. This was essential to achieve a fast and effective system for monitoring the VHF band and detecting its channels. This spectral analyzer was developed using the FFT method with 32,768 points, thus ensuring good characterization of the 3 MHz bandwidth. This analyzer had an update rate of 0.5 s, which is ideal for detecting voice channels.

After this, the algorithms were designed using two methods: Energy Detection and Waveform-Based Detection. Waveform-Based Detection was based on two machine learning models, SVM and KNN. The results of the tests with ED, using a predefined detection threshold, showed that although this model is the least demanding from a computational point of view, which is a significant advantage for low-cost systems such as the one we intend to implement, its detection capability proved to be limited at short distances. The $P_{d}$ was 100% at 200 m but began to decline sharply from that distance, failing to detect any communication at 500 m. For the Waveform-Based Detection method with the KNN model, the detection performance was better than ED for longer distances, where at 700 m it achieved a $P_{d}$ of around 23%, and at the remaining distances it was 8%. However, this system cannot be considered for real-time monitoring systems due to its low $P_{d}$. Both the ED method and machine learning with KNN perform excellently in terms of false positives, $P_{f}$. The last model to be tested was SVM for the Waveform-Based Detection method. This is the one with the best performance, as the $P_{d}$ for the distances tested was around 75% for 900 m, and at 1000 m it had a $P_{d}$ of 80%, which is significantly higher than the other two methods analyzed. In the case of $P_{f}$, the performance is worse than ED and KNN but insignificantly since the probability was only 1%.

Therefore, for the implementation of the real-time detection of communication channels in the VHF band, the Waveform-Based Detection method with the SVM model presented the best performance, demonstrating that the detection of maritime communications can assist security forces in assessing situations with possible clandestine causes or risks to national security. One of the relevant aspects that demonstrates the good performance of the developed system is that, in the tests, a 5 W radio was used, and the detection $P_{d}$ at 1000 m was 80%. If extrapolated to a 25 W maritime radio, the maximum range would then be around 2200 m, which is very good for a low-cost system. Although it is important to emphasize that, while the tests were conducted in an open park environment, where controlled conditions ensured reliable validation of the algorithm, and an open sea environment presents different characteristics due to both the sea surface and the atmospheric conditions encountered there, these results remain applicable to the detection of maritime communications in the VHF band. This demonstrates the potential of the system developed to support the surveillance of ports and coastal areas, enabling the rapid detection of unusual communications and assisting authorities in maintaining maritime safety.

## Figures and Tables

**Table 1:** Classification report for the best SVM model.

Class	Precision (%)	Recall (%)	F1-Score (%)	Accuracy (%)
Active	100	100	100	100
Noise	100	100	100	

**Table 2:** Classification report for the best KNN model.

Class	Precision (%)	Recall (%)	F1-Score (%)	Accuracy (%)
Active	100	80	89	88
Noise	78	100	88	

## Data Availability

The datasets presented in this article are not readily available because, it was developed in a project with sensitive evaluation for the datasets created in it. Requests to access the datasets should be directed to info@inov.pt.
